# Dyslipidemia, subclinical inflammation, hepatic cholestasis and endothelial dysfunction in schoolchildren with excess fat: A study from the United Arab Emirates

**DOI:** 10.1371/journal.pone.0210316

**Published:** 2019-01-09

**Authors:** Elhadi H. Aburawi, Sania Al Hamad, Javed Yasin, Lolowa A. Almekhaini, Abdul-Kader Souid

**Affiliations:** 1 Department of Pediatrics, College of Medicine and Health Sciences, UAE University, Alain, United Arab Emirates; 2 Department of Medicine, College of Medicine and Health Sciences, UAE University, Alain, United Arab Emirates; University of Louisville School of Medicine, UNITED STATES

## Abstract

**Background:**

The impact of obesity on cardiovascular health of young children is still to be fully illustrated. This study measured biomarkers for glycemic control, lipid metabolism, systemic inflammation, endothelial dysfunction, and hepatic cholestasis in schoolchildren. Its main purpose was to determine whether metabolic derangements could be detected in young children with excess fat.

**Method:**

This cross-sectional study involved 967 children in the second, sixth, and tenth grades (median age, 7.3, 11.3, and 15.4 years, respectively). Using the International Obesity Task Force interpretation (IOTF) of body-mass-index (BMI), children were stratified as thin (<5th centiles), normal (5th to <85^th^ centiles), overweight (85th to <95^th^ centiles), obese (95th to <98^th^ centiles), or extremely-obese (≥98^th^ centiles). Waist circumference was also measured. Several metabolic determinations were then used as surrogate biomarkers for cardiovascular risks.

**Results:**

Prevalence of BMI≥85^th^ centile among the second graders was 13.1%, sixth graders 42.2%, and tenth graders 33.8%. BMI≥85^th^ centile was associated with a tendency for higher hemoglobin A_1c_ (*p*≥0.160) and higher blood glucose (*p*≥0.197). For the second graders, BMI≥85^th^ centile was associated with higher high-sensitivity C-reactive protein (hs-CRP, *p*<0.001), higher tumor necrosis factor-α (TNF-alpha, *p*<0.001), higher interleukin-6 (IL-6, *p*<0.001), higher soluble intercellular cytoadhesive molecule-1 (sICAM-1), higher triglycerides (*p*≤0.024), and lower high-density lipoprotein (HDL, *p*<0.001). Additionally, for the sixth and tenth graders, BMI≥85^th^ centile was associated with higher gamma-glutamyl transferase (GGT, *p*<0.001). In the sixth graders, BMI≥85^th^ centile was insignificantly changed with sICAM-1 or the soluble vascular cytoadhesive molecule-1 (sVCAM-1).

**Conclusions:**

The studied children with excess fat had increased risks for developing systemic inflammation, dyslipidemia, endothelial dysfunction, cholestasis, and diabetes. These results suggest that metabolic biomarkers should be included in the routine assessment of children with an overweight problem.

## Introduction

Obesity, diabetes, dyslipidemia, hypertension, and metabolic syndrome are well-known causes of cardiovascular disease [1]. Children are especially vulnerable to these adverse events as they are less likely to be engaged in vigorous health promoting, screening and monitoring programs. As described in detail previously [2], there is a steady rise in obesity (including extreme obesity) among Emirati children 3 to 18 years of age. Each year an additional 2.36% of the Emirati schoolchildren become obese and 0.28% become extremely obese. Therefore, measures designed for assessing and reversing childhood overweight are highly needed [2]. Furthermore, monitoring biomarkers for systemic inflammation, endothelial dysfunction, and hepatic cholestasis is necessary for proper assessment of the negative impact of access fat on human health. This study is the first to address adverse metabolic events in Emirati schoolchildren with overweight problems. Its main aim was to highlight the fact that cardiovascular risks could be detected early in children with obesity.

Systemic inflammation is a critical adverse event in obesity. Excess fat causes sustained elevations in common inflammatory biomarkers, such as hs-CRP (high-sensitivity C-reactive protein), IL-6 (interleukin-6), and TNF-alpha (tumor necrosis factor-alpha). Inflammatory molecules are major contributors to insulin resistance, endothelial dysfunction, and atherosclerosis seen in children with overweight problems [3–4]. Soluble ICAM-1 (intercellular cytoadhesive molecule-1) and VCAM-1 (vascular cytoadhesive molecule-1) are monocyte-promoting adherence molecules, which are expressed in response to cytokine-mediated inflammation [5,6]. Similarly, adiponectin is an adipocyte-derived cytokine that improves insulin sensitivity and ameliorates systemic inflammation [6–11].

This study investigated biomarkers for glycemic control (hemoglobin A_1c_ and random blood glucose), lipid metabolism (adiponectin, triglycerides, total cholesterol, high-density lipoprotein [HDL], and low-density lipoprotein [LDL]), systemic inflammation (high-sensitivity hs-CRP, TNF-alpha, and IL-6), endothelial dysfunction (sICAM-1 and sVCAM-1), and hepatic cholestasis (gamma-glutamyl transferase [GGT]) in Emirati schoolchildren. Its mean purpose was to determine whether these biomarkers could detect cardiovascular risks (especially subclinical inflammation, dyslipidemia, and pre-diabetes) in young children with excess fat.

## Materials and methods

This study involved Emirati students attending 25 Alain governmental schools at grades 2 (elementary school), 6 (middle school) and 10 (high school); expected ages were 8, 12 and 16 years, respectively. The study was approved by Alain Medical District Human Research Ethics Committee (AAMD-HREC-2015-3236 15–112) and Abu Dhabi Education Council (ADEC). Written study consent was obtained for each participant; consent was obtained from parents or guardians for all the minors included in this study.

As described in detail previously [1], students were randomly recruited by a systematic sampling design; even-numbered students on the ADEC school list were selected. A validated and age appropriate study health questionnaire was distributed to about 2,000 selected students and the response rate was 48.4%. All consented students (consent was obtained from parents or guardians for all the minors included in this study) had anthropometric measurements (weight, height, and waist circumference) and physical examination by a trained nurse. The weight and height were measured by a digital scale stadiometer. Children were asked to stand straight with their heads, backs, and buttocks vertically aligned to the height gauge; their heights were then taken and rounded to the nearest 0.5 cm. Waist circumference was measured with upstretched tapes, midpoint between the bottom of the rib cage and the tip of the iliac crest [1]. Gender-specific body-mass index (BMI) growth charts (US Centers for Disease Control and Prevention, CDC) were used to identify overweight (BMI ≥85th centile and <95th centile), obese (BMI ≥95th centile and <99th percentile) and extremely obese (BMI ≥99th percentile) children [12]. BMI was also determined according to the International Obesity Task Force (IOTF) and World Health Organization (WHO) criteria: Thin <5th centiles; normal 5th to <85^th^ centiles; overweight 85th to <95^th^ centiles; obese 95th to <98^th^ centiles; and extremely-obese ≥98^th^ centiles [13]. A website was developed to process BMI values according to the IOTF, WHO, and CDC cut-off criteria [14]. As described in detail previously [2], Microsoft Active Server Pages (ASP) were used for data processing and JavaScript was used for data entry checking. Microsoft SQL Server and relational database management system were used for storing and retrieving data pertaining to the website. The ASP program contained the algorithms, and the database contained the tables needed for calculating BMI values and centiles for the three reference methods.

Random blood samples were collected and processed for glucose, hemoglobin A_1c_ (HbA_1c_) low density lipoprotein (LDL), high-density lipoprotein (HDL), triglycerides, total cholesterol, interleukin-6 (IL-6), tumor necrosis factor-α (TNF-alpha), high-sensitivity C reactive protein (hs-CRP), soluble intercellular cytoadhesive molecule-1 (sICAM-1), soluble vascular cytoadhesive molecule-1 (sVCAM-1), adiponectin, and gamma-glutamyl transferase (GGT). Glucose, HbA_1c_, GGT and lipid profile were measured using an automated analyzer Integra 400 Plus (Roche Diagnostics, Mannheim, Germany).

Enzyme linked immunosorbent assays from R&D Systems were used to measure adiponectin (Acrp30 Quantikine, DRP300), IL-6 (Human IL-6 Quantikine HS, HS600B), TNF-alpha (Human TNFα Quantikine, DTA00C), sICAM-1, and sVCAM-1 following the manufacturers’ protocols. Hs-CRP was measured using Synchron Clinical System (UniCel DxC-800) from Beckman Coulter, Inc. (Fullerton, CA, USA).

The statistical analysis was performed using SPSS software version 21.0 (SPSS Inc., Chicago, USA). Data are presented as median, mean, and standard deviation. Multiple groups were compared using Kruskal-Wallis test, as measurements were either not normally distributed or heteroscedastic (unequal variances). *P*<0.05 was considered significant. Effect size (Cohen's *d*) was calculated on SPSS using z-score of the tested variable followed by independent-samples t-test (mean difference); effect sizes were classified as small (*d*   =   0.2), medium (*d*   =   0.5), or large (*d* ≥ 0.8). Raw data are submitted as S1 Dataset and S2 Dataset.

## Results

Nine hundred sixty-seven participants were enrolled in this study. Their characteristics are summarized in Table 1. Cardiovascular risk factors were more prevalent in the fathers than in the mothers. In this study culture, intratribal marriages were common (35.8%), Table 1.

**Table 1:** Student characteristics (n = 967).

*Grade 2*	
No. (%) Age (y) Female Male Waist circumference (cm) Waist-to-height ratio Weight (kg) Height (cm) BMI (kg/m^2^) Overweight (No., %) Obese (No., %) Extremely-obese (No., %)	337 (34.9) 7.4 ± 0.5 (7.3) 183 (54.3) 154 (45.7) 50.8 ± 7.2 (49.0) 0.40 ± 0.05 (0.39) 24.8 ± 6.5 (23.0) 126.1 ± 4.5 (127.0) 15.5 ± 3.4 (14.6) 21 (6.3) 12 (3.6) 11 (3.3)
*Grade 6*	
No. (%) Age (y) Female Male Waist circumference (cm) Waist-to-height ratio Weight (kg) Height (cm) BMI (kg/m^2^) Overweight (No., %) Obese (No., %) Extremely-obese (No., %)	358 (37.0) 11.3 ± 0.6 (11.3) 200 (58.5) 142 (41.5) 64.2 ± 11.4 (62.0) 0.44 ± 0.07 (0.43) 44.5 ± 14.0 (41.9) 144.6 ± 7.8 (145.0) 21.0 ± 5.5 (20.1) 90 (25.1) 35 (9.8) 26 (7.3)
*Grade 10*	
No. (%) Age (y) Female Male Waist circumference (cm) Waist-to-height ratio Weight (kg) Height (cm) BMI (kg/m^2^) Overweight (No., %) Obese (No., %) Extremely-obese (No., %)	272 (28.1) 15.6 ± 0.9 (15.4) 150 (55.1) 122 (44.9) 70.9 ± 13.7 (67) 0.44 ± 0.08 (0.42) 62.5 ± 19.3 (57.0) 161.5 ± 9.0 (161.0) 23.9 ± 6.6 (21.8) 42 (15.4) 26 (9.6) 24 (8.8)
*Maternal variables*, No. (%)	
Hypertension Dyslipidemia Diabetes Excess body fat	51 (5.3) 54 (5.6) 58 (6.0) 39 (4.0)
*Paternal variables*, No. (%)	
Hypertension Dyslipidemia Diabetes Excess body fat	134 (13.8) 105 (10.9) 153 (15.8) 89 (9.2)
*Consanguinity*, No. (%)	346 (35.8)

Values are No. (%) or mean ± SD (with the values in parentheses being median). Some students had missing data; thus, the percentiles are based on available data. Overweight (BMI, 85th to <95th), obese (BMI, 95th to <98th), and extremely-obese (BMI, ≥98th) are based on IOTF interpretation of BMI.

Chi-squared test was used to analyze whether any of the family variables correlate with excess body fat (IOTF classification of BMI as “thin or normal” versus “overweight, obese or extremely-obese”) in the studied children. Pearson Chi-square (asymptotic significance, 2-sided) <0.05 was considered significant. Only father or mother with diabetes (p≤0.001) and father with excess body fat (p = 0.004) significantly correlated with children with excess body fat.

**Fig 1:**
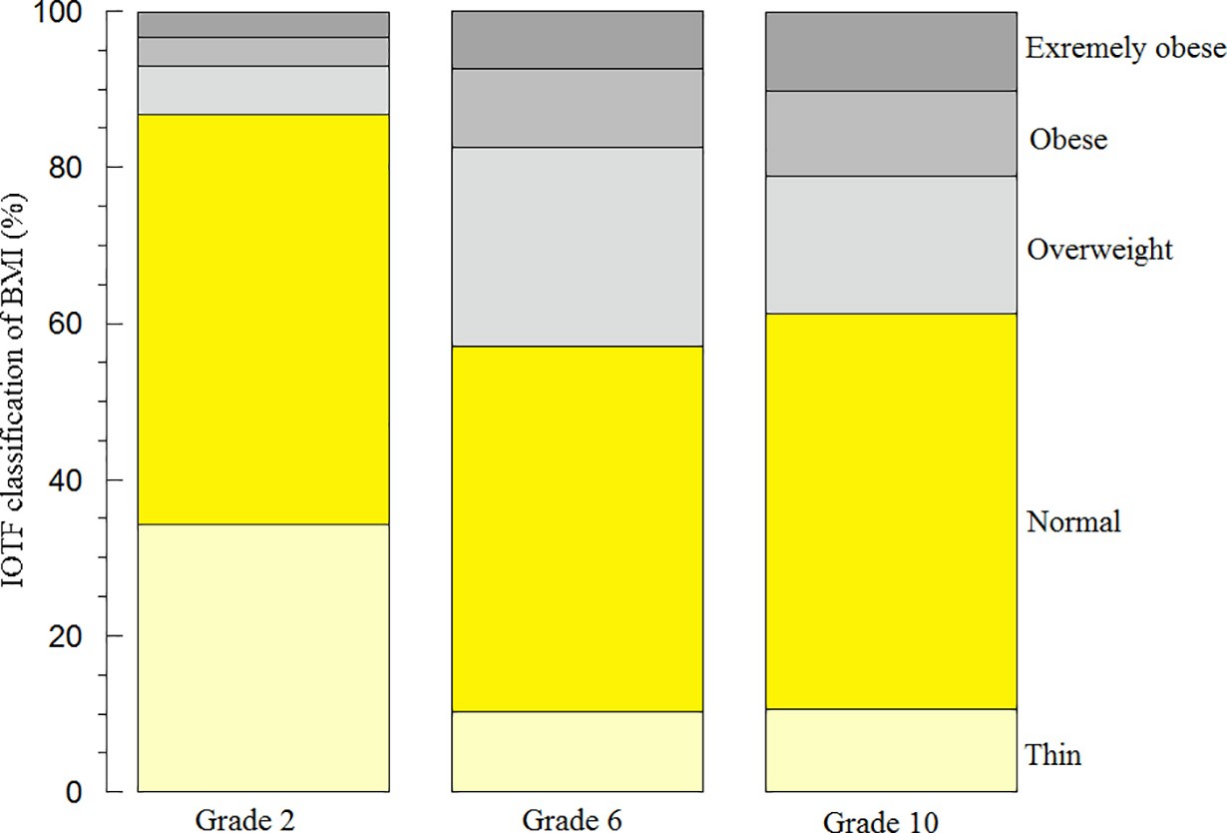
BMI of the studies children per IOTF, WHO, and CDC classifications. The values of BMI were classified as thin, normal, overweight, obese, or extremely obese according to IOTF (upper panel), WHO (middle panel), and CDC (lower panel) criteria.

**Fig 2:**
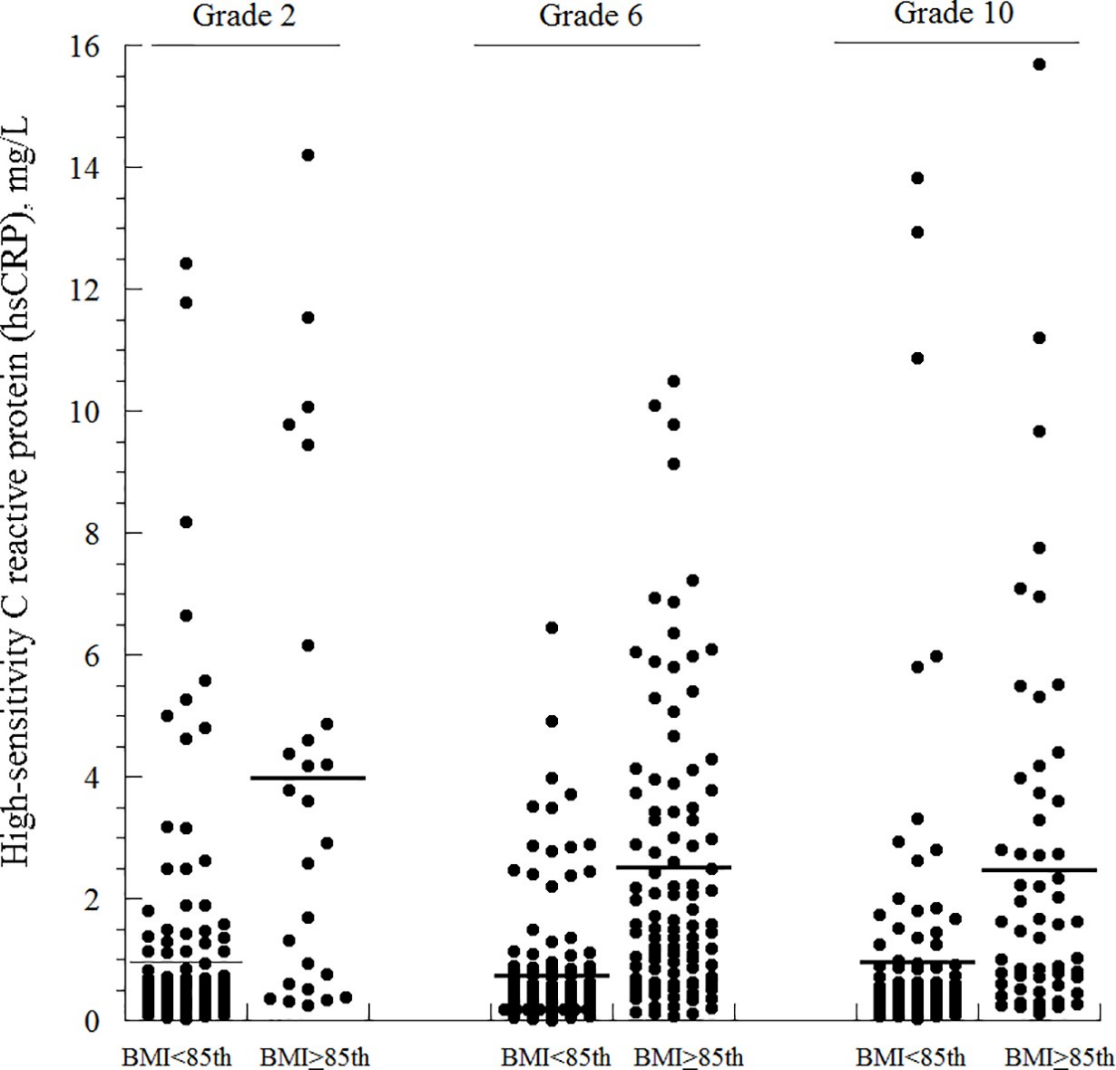
Dot plots of representative biomarkers (hs-CRP, HDL, and GGT) in the studied children as functions of grade and IOTF-BMI (<85^th^ centile versus ≥85^th^ centile). Horizontal lines are mean. One student (grade 10, BMI >85^th^ centile) with a GGT value of 188 U/L is not shown in the plot.

Using IOTF interpretation of BMI, the prevalence of BMI ≥85^th^ centile among the second graders was 13.1%, among the sixth graders was 42.2%, and among the tenth graders was 33.8% (Fig 1 and Table 1). Similar frequencies were also obtained using the CDC and WHO criteria (Fig 1). The prevalence of BMI ≥85^th^ centile was similar among boys and girls. Thus, childhood obesity was common and progressed with time. Representative biomarkers (hs-CRP, HDL, and GGT) in the studied children as functions of grade and IOTF-BMI (<85^th^ centile versus ≥85^th^ centile) are shown in Fig 2.

Compared to second graders with IOTF-BMI <85^th^ centile, those with IOTF-BMI ≥85^th^ centile had higher hs-CRP (*p*<0.001, Cohen's *d =* 1.190), higher IL-6 (*p*<0.001, *d =* 1.096), higher TNF-alpha (*p*<0.001, *d* = 1.100), higher sICAM (*p* = 0.024, *d* = 0.592), higher LDL (*p* = 0.015, *d* = 0.781), and lower HDL (*p*<0.001, *d* = 0.750), Table 2.

**Table 2:** Results of the measured biomarkers as functions of grade and IOTF classification of BMI.

*Grade 2 (169 students consented to blood testing)*							
	Thin (n = 55)	Normal (n = 88)		Overweight, obese, or extremely-obese (n = 26)		*P*	
Hemoglobin A_1c_ (%), n = 165	5.1 ± 0.4 (5.1)	5.1 ± 0.4 (5.1)		5.3 ± 0.3 (5.3)		0.097	
Random blood glucose (mmol/L), n = 169	5.0 ± 1.1 (4.9)	5.0 ± 1.2 (4.7)		5.3 ± 1.0 (5.2)		0.079	
hs-CRP (mg/L), n = 169	0.9 ± 1.9 (0.3)	1.0 ± 1.8 (0.5)		4.0 ± 4.0 (3.3)		**<0.001**	
Total cholesterol (mmol/L), n = 169	4.1 ± 0.7 (4.0)	4.1 ± 0.6 (4.1)		4.3 ± 0.7 (4.3)		0.243	
Triglyceride (mmol/L), n = 169	0.8 ± 0.3 (0.7)	0.9 ± 0.5 (0.7)		1.0 ± 0.5 (0.9)		0.057	
HDL (mmol/L), n = 169	1.4 ± 0.4 (1.4)	1.5 ± 0.3 (1.5)		1.2 ± 0.3 (1.3)		**<0.001**	
LDL (mmol/L), n = 169	2.5 ± 0.5 (2.4)	2.5 ± 0.6 (2.5)		3.0 ± 0.8 (2.8)		**0.015**	
Interleukin 6 (pg/mL), n = 101	4.2 ± 2.6 (3.5)	3.0 ± 1.6 (2.3)		6.0 ± 3.8 (5.7)		**<0.001**	
TNF-alpha (pg/mL), n = 101	6.0 ± 2.8 (5.0)	5.0 ± 1.8 (4.4)		8.4 ± 4.1 (7.8)		**<0.001**	
Adiponectin (μg/mL),n = 101	5.2 ± 3.1 (4.2)	6.3 ± 3.9 (5.5)		5.4 ± 3.2 (4.3)		<0.177	
sICAM-1 (ng/mL), n = 101	231 ± 40 (232)	263 ± 77 (254)		309 ± 85 (299)		**0.024**	
sVCAM-1 (ng/mL), n = 101	590 ± 109 (608)	633 ± 130 (603)		622 ± 136 (609)		0.894	
GGT (U/L), n = 101	19.5 ± 5.7 (21.0)	22.2 ± 3.5 (22.0)		23.2 ± 5.8 (22.5)		0.668	
*Grade 6 (225 students consented to blood testing**)*							
	Thin (n = 22)	Normal (n = 104)	Overweight (n = 60)		Obese/ extremely-obese (n = 39)		*P*
Hemoglobin A_1c_ (%), n = 225	5.2 ± 0.3 (5.2)	5.3 ± 0.4 (5.4)	5.3 ± 0.5 (5.4)		5.2 ± 0.3 (5.3)		0.197
Random blood glucose (mmol/L), n = 225	4.8 ± 0.7 (4.8)	4.9 ± 0.6 (4.8)	5.1 ± 0.8 (4.9)		5.3 ± 1.0 (5.0)		0.061
hs-CRP (mg/L), n = 225	0.4 ± 0.6 (0.2)	0.8 ± 1.1 (0.4)	2.2 ± 2.3 (1.4)		3.0 ± 2.4 (2.4)		**<0.001**
Total cholesterol (mmol/L), n = 225	4.0 ± 0.5 (4.0)	4.2 ± 0.7 (4.2)	4.2 ± 0.7 (4.1)		4.3 ± 0.9 (4.1)		0.481
Triglyceride (mmol/L), n = 225	0.8 ± 0.3 (0.7)	0.9 ± 0.4 (0.7)	1.0 ± 0.4 (0.9)		1.2 ± 0.6 (1.2)		**<0.001**
HDL (mmol/L), n = 225	1.4 ± 0.3 (1.5)	1.5 ± 0.4 (1.5)	1.3 ± 0.3 (1.2)		1.2 ± 0.2 (1.1)		**<0.001**
LDL (mmol/L), n = 225	2.3 ± 0.5 (2.4)	2.5 ± 0.6 (2.5)	2.7 ± 0.6 (2.7)		2.7 ± 0.8 (2.6)		0.051
Interleukin 6 (pg/mL), n = 105	-	3.0 ± 1.3 (2.6)	4.4 ± 2.2 (3.9)		5.8 ± 2.4 (5.7)		**<0.001**
TNF-alpha (pg/mL), n = 105	-	4.8 ± 1.3 (4.4)	6.4 ± 2.8 (5.6)		8.0 ± 3.1 (7.9)		**0.001**
Adiponectin (μg/mL) n = 105	-	5.2 ± 2.2 (4.8)	4.7 ± 2.2 (4.2)		4.5 ± 2.4 (4.1)		0.457
sICAM-1 (ng/mL), n = 105	-	274 ± 77 (273)	293 ± 101 (287)		342 ± 88 (326)		**0.031**
sVCAM-1 (ng/mL), n = 105	-	616 ± 97 (598)	631 ± 125 (610)		647 ± 96 (659)		0.752
GGT (U/L), n = 105	-	21.2 ± 4.1 (21.0)	24.7 ± 5.9 (23.0)		27.5 ± 6.8 (25.5)		**<0.001**
*Grade 10 (170 students consented to blood testing)*							
	Thin (n = 20)	Normal (n = 92)	Overweight (n = 25)		Obese/ extremely-obese (n = 33)		*P*
Hemoglobin A_1c_ (%), n = 170	5.0 ± 0.5 (5.2)	5.2 ± 0.4 (5.2)	5.3 ± 0.4 (5.3)		5.3 ± 0.6 (5.2)		0.196
Random blood glucose (mmol/L), n = 170	5.3 ± 1.8 (4.7)	5.0 ± 0.9 (4.8)	5.3 ± 1.1 (5.1)		5.6 ± 2.1 (5.1)		0.160
hs-CRP (mg/L), n = 170	1.0 ± 2.4 (0.2)	0.9 ± 2.1 (0.3)	1.2 ± 1.6 (0.5)		3.5 ± 3.5 (2.7)		**<0.001**
Total cholesterol (mmol/L), n = 170	4.3 ± 1.6 (3.7)	4.0 ± 0.7 (3.8)	3.9 ± 0.6 (3.7)		4.4 ± 1.3 (4.3)		0.189
Triglyceride (mmol/L), n = 170	0.9 ± 0.9 (0.7)	1.6 ± 7.8 (0.7)	1.1 ± 0.6 (0.9)		1.5 ± 1.7 (1.1)		**0.001**
HDL (mmol/L), n = 170	1.5 ± 0.5 (1.5)	1.5 ± 0.4 (1.4)	1.3 ± 0.3 (1.3)		1.2 ± 0.4 (1.1)		**<0.001**
LDL (mmol/L), n = 170	2.6 ± 1.3 (2.3)	2.5 ± 0.7 (2.5)	2.4 ± 0.6 (2.4)		2.8 ± 0.8 (2.9)		0.125
Interleukin 6 (pg/mL), n = 114	4.7 ± 3.0 (4.5)	3.2 ± 2.1 (2.5)	3.3 ± 1.8 (2.4)		5.3 ± 2.4 (5.0)		**<0.001**
TNF-alpha (pg/mL), n = 114	6.7 ± 3.8 (6.2)	5.1 ± 2.2 (4.4)	5.4 ± 1.3 (3.0)		7.4 ± 2.5 (6.9)		**<0.001**
Adiponectin (μg/mL), n = 114	3.9 ± 1.6 (4.0)	4.2 ± 1.8 (4.0)	3.0 ± 2.6 (5.6)		3.5 ± 2.0 ()		**0.040**
sICAM-1 (ng/mL), n = 114	215 ± 89 (189)	223 ± 42 (221)	216 ± 47 (210)		235 ± 60 (220)		0.610
sVCAM-1 (ng/mL), n = 114	578 ± 184 (580)	540 ± 118 (529)	561 ± 105 (541)		590 ± 144 (555)		0.497
GGT (U/L), n = 114	16.5 ± 3.3 (17.0)	22.0 ± 8.3 (21.0)	25.8 ± 7.4 (24.0)		32.4 ± 19.2 (25.5)		**<0.001**

Values are mean ± SD; values in parentheses are median. The values of *p* are Kruskal Wallis test (asymptotic significance). Hs-CRP, high-sensitivity C reactive protein; HDL, high-density lipoprotein; LDL, low-density lipoprotein; TNF-α, tumor necrosis factor-alpha; sICAM-1, soluble intercellular adhesion molecule-1; sVCAM-1, soluble vascular cell adhesion molecule-1; GGT, gamma-glutamyl transferase.

Total cholesterol ≥5.18 mmol/L requires treatment. Triglyceride ≥1.5 mmol/L in patients 10–19 y are high. HDL is considered normal if >1.17 mmol/L and low if <0.91 mmol/L. Normal LDL is <2.85 mmol/L.

Children were stratified as thin (<5th percentile), normal (5th to <85th), overweight (85th to <95th), obese (95th to <98th), or extremely-obese (≥98th), using the International Obesity Task Force (IOTF) interpretation of body-mass-index (BMI).

Analysis of the five IOTF groups of BMI (underweight, normal, overweight, obese, and extremely-obese) was performed on combined grades using Kruskal-Wallis test; all the laboratory biomarkers were significantly different among the groups (p<0.038), except the VCAM-1 (p<0.742).

Similarly, the sixth graders with BMI ≥85^th^ centile had higher hs-CRP (*p*<0.001, *d* = 1.769), higher triglycerides (*p*<0.001, *d* = 0.240), higher LDL (*p* = 0.051, *d* = 0.215), higher IL-6 (*p*<0.001, *d* = 1.653), higher TNF-alpha (*p* = 0.001, *d* = 1.922), higher sICAM (*p* = 0.031, *d* = 33.880), higher GGT (*p*<0.001, *d* = 4.456), and lower HDL (*p*<0.001, *d* = 0.243), Table 2. The tenth graders with BMI ≥85^th^ centile had higher hs-CRP (*p*<0.001, *d* = 1.530), higher triglycerides (*p* = 0.001, *d* = 0.153), higher IL-6 (*p*<0.001, *d* = 1.097), higher TNF-alpha (*p*<0.001, *d* = 1.243), higher GGT (*p*<0.001, *d* = 7.992), lower HDL (*p*<0.001, *d* = 0.253), and higher adiponectin (*p*<0.040, *d* = 0.942). sICAM and sVCAM-1 did not significantly change in this age group (Table 2). Thus, these biomarkers detected significant metabolic derangements in young children with overweight problems. It is worth noting that hemoglobin A_1c_ and random blood glucose were higher in children with BMI≥85^th^ centile, but the differences did not reach the statistical significance (Table 2).

Positive correlations were observed only between the inflammatory biomarkers; hs-CRP versus IL6 (*R*^2^ = 0.875), hs-CRP versus TNF-alpha (*R*^2^ = 0.888), and IL6 versus TNF-alpha (*R*^2^ = 0.933). hs-CRP did not correlate with HDL (*R*^2^ = 0.047) or hemoglobin A_1c_ (*R*^2^ = 0.000). Similarly, there were no significant correlations between HDL, adiponectin, sICAM-1, sVCAM-1, and GGT (*R*^2^≤0.1).

Table 3. summarizes the measurements of ‘waist circumference’ and ‘waist-to-height ratio’, given as functions of IOTF-BMI and age. Both parameters significantly increased in children with overweight, obesity, or extreme-obesity (*p*<0.001). Thus, both waist circumference and waist-to-height ratio (abdominal or central obesity) should be used to measure the stage of obesity.

**Table 3:** Waist circumference and waist-to-height ratio as functions of IOTF-BMI and age.

*Age*	*Thin*	*Normal*	*Overweight*	*Obese*	*Extremely-obese*	*P*
	**Waist Circumference (cm)**					
6 y	47.2±3.6 (47.0)	50.3±3.9 (49.5)	54.2±3.7 (54.0)	62.8±3.3 (64.0)	64.0±12.7 (64.0)	<0.001
7 y	47.1±4.1 (47.0)	50.6±5.9 (50.0)	57.5±3.4 (57.0)	64.5±3.3 (65.5)	71.2±11.6 (72.5)	<0.001
8 y	41.5±2.1 (41.5)	48.6±4.8 (48.0)	-	-	71.7±8.7 (74.0)	<0.001
10 y	51.9±3.8 (51.5)	51.9±5.8 (58.0)	67.1±4.6 (66.0)	72.6±2.6 (73.0)	81.9±15.6 (80.0)	<0.001
11 y	52.7±5.3 (52.0)	58.4±4.8 (58.0)	69.8±7.6 (69.0)	78.7±9.0 (79.0)	82.3±14.9 (86.0)	<0.001
14 y	60.3±8.1 (58.5)	64.3±6.3 (63.0)	69.2±4.8 (69.0)	87.3±7.1 (86.0)	-	<0.001
15 y	61.1±8.5 (59.0)	63.5±5.8 (63.0)	74.3±7.4 (74.5)	85.5±6.8 (85.0)	95.9±8.8 (95.5)	<0.001
16 y	63.0±4.2 (64.5)	65.8±8.7 (63.0)	79.7±8.4 (80.5)	84.9±13.8 (84.0)	107.7±2.1 (107.0)	<0.001
	**Waist-to-Height Ratio**					
6 y	0.38±0.03 (0.38)	0.40±0.03 (0.39)	0.43±0.03 (0.43)	0.48±0.03 (0.49)	0.49±0.10 (0.49)	<0.001
7 y	0.40±0.03 (0.38)	0.40±0.05 (0.40)	0.45±0.03 (0.45)	0.49±0.03 (0.50)	0.54±0.07 (0.55)	<0.001
8 y	0.33±0.01 (0.33)	0.38±0.04 (0.37)	-	-	0.55±0.06 (0.56)	<0.001
10 y	0.37±0.03 (0.37)	0.41±0.03 (0.41)	0.47±0.04 (0.47)	0.50±0.03 (0.51)	0.56±0.10 (0.58)	<0.001
11 y	0.38±0.04 (0.37)	0.40±0.07 (0.40)	0.47±0.05 (0.46)	0.53±0.07 (0.53)	0.55±0.09 (0.57)	<0.001
14 y	0.38±0.04 (0.38)	0.40±0.03 (0.40)	0.44±0.04 (0.43)	0.54±0.05 (0.51)	-	<0.001
15 y	0.38±0.05 (0.37)	0.39±0.03 (0.39)	0.46±0.04 (0.46)	0.52±0.03 (0.53)	0.57±0.04 (0.58)	<0.001
16 y	0.40±0.03 (0.38)	0.41±0.05 (0.40)	0.47±0.03 (0.48)	0.52±0.07 (0.50)	0.65±0.02 (0.54)	<0.001

Values are mean ± SD; values in parentheses are median. The values of p are Kruskal Wallis test (asymptotic significance).

Children were stratified as thin (<5th percentile), normal (5th to <85th), overweight (85th to <95th), obese (95th to <98th), or extremely-obese (≥98th), using the International Obesity Task Force (IOTF) interpretation of body-mass-index (BMI).

## Discussion

This study reports on the status of glycemic control, lipid metabolism, systemic inflammation, endothelial dysfunction, and hepatic cholestasis in schoolchildren with excess fat. The results show that significant metabolic derangements are evident in elementary school children with overweight problems. Thus, excess fat negatively impacts the cardiovascular health of young children and prompt interventions are necessary.

Waist-to-height ratio, waist circumference (measured at the midpoint between the bottom of the rib cage and the tip of the iliac crest), and waist-to-hip ratio are simple indicators of cardiovascular risk in persons with obesity. While BMI is the traditional measure of overweight or obese, it is not a good indicator of health risk assessment.

Prevention of childhood obesity requires multidisciplinary efforts, which include regular monitoring during all healthcare visits. Management of obesity necessitates prompt enrolment in a structured program that endorses slow weight loss (good diet and regular exercise) through a family-based behavioral treatment. Psychological problems should be well managed before children begin a weight loss program. Five percent reduction in BMI can significantly improve the biochemical derangement associated with obesity. Estimated caloric need for 4 to 6 year-old children is 80 to 90 kcal/kg/day and for 7 to 10 year-old children is 70 to 80 kcal/kg/day (weight gain, 3–5 g/day). Very-low-calorie diets (e.g., 1,000 calories/day) can lead to nutritional deficiencies and should be avoided.

The studied biomarkers detected significant metabolic derangements in children with overweight problems, which were evident in early childhood. Therefore, the excess fat in these young children increased their risks for systemic inflammation, dyslipidemia, endothelial dysfunction, hepatic cholestasis, and dysglycemia. Thus, routine workup for childhood obesity should include screening for dyslipidemia, diabetes (including insulin resistance), subclinical inflammation, fatty liver, hypertension, obstructive sleep apnea, metabolic syndrome, and polycystic ovarian syndrome. In addition, genetic testing should be offered for children who have extreme obesity [15]. We show here that the biochemical profile that uses hs-CRP, IL-6, TNF-alpha, Hb A_1c_, adiponectin, sICAM-1, sVCAM-1, and GGT is helpful. The lack of correlations between the inflammatory biomarkers and the biomarkers of dyslipidemia and hepatic cholestasis (GGT) may suggest that these obesity-associated derangements are independent. The development of obesity needs to be carefully monitored and promptly treated in children and adolescents. The use of biomarkers may help the child and parents to appreciate the seriousness of obesity and encourage them to enroll in a proper weight reduction program.

Children with obesity usually consume high calories from nutrient-poor and calorie-dense foods and drinks. They usually have long screen-time and participate less in vigorous exercises. Reversing these habits requires effective counseling and motivations at home and at school. The impact of prescribing drugs that promote weight loss (e.g., orlistat) is still to be illustrated. Bariatric surgery is considered only for adolescents with a sexual maturity rating of 4 to 5, and BMI of 40 kg/m^2^ or BMI of 35 kg/m^2^ plus obesity-associated complications.

Obesity and its related disorders are associated with a reduction in the lifespan of about 12 years. In 2008, The Obesity Society recognized obesity as a “disease” [16]. In 2013, the American Medical Association recognized obesity as a chronic complex condition requiring intervention [17]. The 2011 Expert Panel on Integrated Guidelines for Cardiovascular Health and Risk Reduction in Children and Adolescents recommends lipid screening (nonfasting LDL or fasting lipid panel) between 9 and 11 years and a second screening between 18 and 21 years [18]. Other recommendations include breast-feeding, low intake of saturated fat beginning at 1 year of age, stopping exposure to tobacco, and regular physical activity. Nearly 1 in 3 children screened for high cholesterol at 9 to 11 years have borderline or high levels, thereby increasing their risk for cardiovascular disease. Statin is recommended for LDL >4.9 mmol/L, LDL >4.1 mmol/L with positive family history, or LDL >3.4 mmol/L with positive diabetes. Children with total cholesterol >5.18 mmol/L, HDL <1.17 mmol/L, and LDL >3.37 mmol/L are at risk of early coronary artery disease. More recently, the US Preventive Services Task Force statement issued the following statement: “The current evidence is insufficient to assess the balance of benefits and harms of screening for lipid disorders in children and adolescents younger than 20 years. If the lipid screening is offered as a service by practitioners, patients (and families) should understand the uncertainty about the balance of benefits and harms.” [19]

Obesity imposes a cluster of subclinical inflammation and endothelial dysfunction. Reduced levels of adiponectin in obesity have been shown to promote inflammatory cytokine-induced expression of cytoadhesive molecules [7]. These results are consistent with our findings of excess fat is associated with decreased adiponectin. In one study, sICAM-1 and sVCAM-1 were measured in children (mean age, 15 y) with obesity [20]. Both adhesive molecules were significantly higher in children with obesity compared to healthy children (sICAM-1: 314 ± 61 ng/mL versus. 265 ± 55 ng/mL; sVCAM-1: 514 ± 187 versus 408 ± 76 ng/mL). sICAM-1 was dependent on BMI and sVCAM-1 was dependent on total cholesterol [20]. The authors concluded “endothelial activation appears in these children” [20]. This current study supports the use of metabolic biomarkers in children with overweight problems. As previously noted, programs that endorse regular exercise and diet modifications, especially in the genetically most susceptible children are highly warranted [21–24].

Significant childhood metabolic derangements are evident in the presence of overweight problems. This finding is consistent with previous regional [1, 2] and international [25, 26] studies, and support current understanding of the ‘childhood origin of adult diseases’. The clinical use of biomarkers of systemic inflammation, dyslipidemia, dysglycemia, liver disease, endothelial dysfunction, and fat metabolism to monitor progress of the adverse events of access body fat is highly encouraged.

In conclusion, in this study, cardiovascular risks were investigated in school children using a set of biomarkers for systemic inflammation, glycemic control, dyslipidemia, endothelial function, and hepatic cholestasis. Significant biomarkers of inflammation (hsCRP, IL-6, and TNF-alpha) and endothelial dysfunction (sICAM-1 and sVCAM-1) are present in young children with obesity. Prospective studies are necessary to investigate the usefulness of these biochemical markers for the proper clinical care of these patients.

## Supporting information

S1 Dataset Raw Data. (XLSX)

S2 Dataset Values interpretation. (XLSX)

## Abbreviations

BMI: body-mass-index
CDC: Centers for Disease Control and Prevention
GGT: gamma-glutamyl transferase
IL-6: interleukin-6
IOTF: International Obesity Task Force interpretation
HbA1c: hemoglobin A_1c_
HDL: high-density lipoprotein
hs-CRP: higher high-sensitivity C-reactive protein
LDL: low-density lipoprotein
sICAM-1: soluble intercellular cytoadhesive molecule-1
sVCAM-1: soluble vascular cytoadhesive molecule-1
TNF-alpha: tumor necrosis factor-alpha
WHO: World Health Organization

## References

[1] Aburawi EH, AlKaabi J, Zoubeidi T, Shehab A, Lessan N, Al Essa A, et al Subclinical inflammation and endothelial dysfunction in young patients with diabetes: A study from United Arab Emirates. PLoS One. 2016 7 26;11(7):e0159808 10.1371/journal.pone.0159808 27459718 PMC4961363

[2] AlBlooshi A, Shaban S, AlTunaiji M, Fares N, AlShehhi L, AlShehhi H, et al Increasing obesity rates in school children in United Arab Emirates. Obes Sci Pract. 2016;2:196–202. 10.1002/osp4.37 27818779 PMC5074293

[3] Libby P. Inflammation in atherosclerosis. Nature. 2002;420: 868–874. 10.1038/nature01323 12490960

[4] Tilg H, Moschen AR. Adipocytokines: mediators linking adipose tissue, inflammation and immunity. Nat Rev Immunol. 2006;6: 772–783. 10.1038/nri1937 16998510

[5] Okuda R, Matsushima H, Aoshiba K, Oba T, Kawabe R, Honda K, et al Soluble intercellular adhesion molecule-1 for stable and acute phases of idiopathic pulmonary fibrosis. Springer plus 2015;4: 657 10.1186/s40064-015-1455-z 26543791 PMC4628606

[6] Ouchi N, Kihara S, Arita Y, Maeda K, Kuriyama H, Okamoto Y, et al Novel modulator for endothelial adhesion molecules: adipocyte-derived plasma protein adiponectin. Circulation 1999;100: 2473–2476. . 10604883 10.1161/01.cir.100.25.2473

[7] Ouchi N, Kihara S, Arita Y, Okamoto Y, Maeda K, Kuriyama H, et al Adiponectin, an adipocyte-derived plasma protein, inhibits endothelial NF-kappaB signaling through a cAMP-dependent pathway. Circulation 2000;102: 1296–1301. . 10982546 10.1161/01.cir.102.11.1296

[8] Ebrahimi-Mamaeghani M, Mohammadi S, Arefhosseini SR, Fallah P, Bazi Z. Adiponectin as a potential biomarker of vascular disease. Vascular Health Risk Manag 2015;16: 55–70. 10.2147/VHRM.S48753 PMC4303398 25653535

[9] Byun SH, Kwon EB, Kim SY. The relationship between serum adiponectin and inflammatory cytokines in obese Korean juveniles. Korean J Pediatr. 2014;57: 533–537. 10.3345/kjp.2014.57.12.533 25653687 PMC4316597

[10] Stefan N, Bunt JC, Salbe AD, Funahashi T, Matsuzawa Y, Tataranni PA. Plasma adiponectin concentrations in children: relationships with obesity and insulinemia. J Clin Endocrinol Metab. 2002;87: 4652–4656. 10.1210/jc.2002-020694 12364452

[11] Nigro E, Scudiero O, Monaco ML, Palmieri A, Mazzarella G, Costagliola C, et al New insight into adiponectin role in obesity and obesity-related diseases. Biomed Res Int. 2014;2014: 658913 10.1155/2014/658913 25110685 PMC4109424

[12] Kuczmarski RJ, Ogden C, Guo S, Grummer-Strawn LM, Flegal KM, Mei Z, et al 2000 CDC Growth Charts for the United States: methods and development. Vital Health Stat 11. 2002 5;(246): 1–190. . 12043359

[13] de Onis M, Onyango AW, Borghi E, Siyam A, Nishida C, Siekmann J. Development of a WHO growth reference for school-aged children and adolescents. Bull World Health Organ. 2007 9;85(9): 660–667. 10.2471/BLT.07.043497 . 18026621 PMC2636412

[14] http://cmhsweb.uaeu.ac.ae/childbmicalculator. Accessed Dec. 2017.

[15] da Fonseca ACP, Mastronardi C, Johar A, Arcos-Burgos M, Paz-Filho G. Genetics of non-syndromic childhood obesity and the use of high-throughput DNA sequencing technologies. J Diabetes Complications. 2017(10): 1549–1561. 10.1016/j.jdiacomp.2017.04.026 28735903

[16] Allison DB, Downey M, Atkinson RL, Billington CJ, Bray GA, Eckel RH, et al Obesity as a disease: a white paper on evidence and arguments commissioned by the Council of the Obesity Society. Obesity (Silver Spring). 2008;16: 1161–77. 10.1038/oby.2008.231 18464753

[17] Kyle TK, Dhurandhar EJ, Allison DB. Regarding Obesity as a Disease: Evolving Policies and Their Implications. Endocrinol Metab Clin North Am. 2016;45: 511–20. 10.1016/j.ecl.2016.04.004 27519127 PMC4988332

[18] Expert Panel on Integrated Guidelines for Cardiovascular Health and Risk Reduction in Children and Adolescents; National Heart, Lung, and Blood Institute. Expert panel on integrated guidelines for cardiovascular health and risk reduction in children and adolescents: summary report. Pediatrics. 2011;128 Suppl 5: S213–56. 10.1542/peds.2009-2107C 22084329 PMC4536582

[19] US Preventive Services Task Force, Bibbins-Domingo K, Grossman DC, Curry SJ, Davidson KW, Epling JW Jr, García FA, et al Screening for Lipid Disorders in Children and Adolescents: US Preventive Services Task Force Recommendation Statement. JAMA. 2016;316: 625–33. 10.1001/jama.2016.9852 27532917

[20] Glowinska B, Urban M, Peczynska J, Florys B. Soluble adhesion molecules (sICAM-1, sVCAM-1) and selectins (sE selectin, sP selectin, sL selectin) levels in children and adolescents with obesity, hypertension, and diabetes. Metabolism. 2005;54: 1020–6. 10.1016/j.metabol.2005.03.004 16092051

[21] Skrypnik D, Ratajczak M, Karolkiewicz J, Mądry E, Pupek-Musialik D, Hansdorfer-Korzon R, et al Effects of endurance and endurance-strength exercise on biochemical parameters of liver function in women with abdominal obesity. Biomed Pharmacother. 2016;80: 1–7. 10.1016/j.biopha.2016.02.017 27133033

[22] Szulińska M, Skrypnik D, Michałowska J, Bogdański P. Non-pharmacological modification of endothelial function: An important lesson for clinical practice. Postepy Hig Med Dosw (online). 2018;72: 89–100. 10.5604/01.3001.0011.5963

[23] Szulińska M, Stępień M, Kręgielska-Narożna M, Suliburska J, Skrypnik D, Bąk-Sosnowska M, et al Effects of green tea supplementation on inflammation markers, antioxidant status and blood pressure in NaCl-induced hypertensive rat model. Food Nutr Res. 2017;61: 1295525 10.1080/16546628.2017.1295525 28326006 PMC5345575

[24] Skrypnik K, Suliburska J, Skrypnik D, Pilarski L, Reguła J. Bogdański P. The genetic basis of obesity complications. Acta Sci. Pol. Technol. Aliment. 2017;16: 83–91. 10.17306/J.AFS.2017.0442. 28362475

[25] Kumar S, Kelly AS. Review of Childhood Obesity: From Epidemiology, Etiology, and Comorbidities to Clinical Assessment and Treatment. Mayo Clin Proc. 2017;92: 251–265. 10.1016/j.mayocp.2016.09.017 28065514

[26] Nehus E, Mitsnefes M. Childhood Obesity and the Metabolic Syndrome. Pediatr Clin North Am. 2019;66: 31–43. 10.1016/j.pcl.2018.08.004 30454749

